# Extracts from a Letter of Mr. Kinning, Assistant Surgeon Royal Artillery, to Dr. Rollo, Dated Gibraltar, December 8, 1804, with a Continuation to the 15th of the Same Month

**Published:** 1805-03-01

**Authors:** 


### Medical and Phyfical Journal.

VOL. XIII.] MARCH 1, 1805. [NO. LXXIII.

*Printed for R. PHILLIPS, by W. Thorne, Red Lion Court, Fleet Street, London.*

### *Extracts from a Letter of* Mr. KINNING, *Assistant Surgeon Royal Artillery, to* Dr. ROLLO, *dated Gibraltar, December* 8, 1804, *with a Continuation to the* 15*th of the same Month.*

"SIR,

"ENCLOSED are eight cases of the malignant fever of Gibraltar; one of them was taken by Mr. Bell, who assisted me for some time in this arduous duty. These patients I have seen even six times a day, to enable me to give you full information of the disorder and treatment, which has been varied. I conceived it necessary to make but two daily reports, judging them sufficient to explain to you the uncertainty of the termination and the insidiousness of the disease.

"From the 12th ult. I took the entire charge of our sick, which were then reduced to twenty-three fevers, as you will perceive by the weekly reports. Since then the number of deaths have been fewer in proportion (even reckoning those only with the strongest marked symptoms of the disease) which I attribute to my having sufficient time to attend to so small a number.

"On Sept. 30, I had charge of 196 patients in the hospital, about thirty of them with sore eyes, &c. not confined to bed, (the others were) all in an excellent hospital in the south part of the Rock, that I recommended for them. At this period, Mr. B. was attending a few cases in town, that could not be removed, and also the women, &c. The garrison could not afford me more assistance, consequently we lost many, as I was inadequate to pay the necessary attention to so many sick. The disease is now exactly similar to what it was when first observed, probably cold weather will make the symptoms milder. The season as yet has been very moderate, indeed more so than usual, only three days rain. And also I conceive that many, who have not yet had the disorder would entirely resist its influence. These reasons promise a speedy termination of it, if government will continue to prevent strangers landing for some time longer.

"Until November 19, from the commencement of this dreadful malady, scarcely an instance occurred that was not of the contagious fever; but as soon as our men returned from camp, and occupied the casements in the Orange bastion, ophthalmia again appeared with its usual virulence.

On October 1, I had tents pitched for the reception of sick women and children, near the hospital, from which they were supported, also four hospital tents for convalescents, to ease the hospital, as it was exceedingly crowded.

"The Medical Staff have recommended precautions respecting the disease, so that in pursuance of this day's general orders, all the men who have not had the present epidemic fever, will be encamped to morrow, and to be kept in strict quarantine; one hundred and twenty of the Artillery will be encamped at Willis, and thirty Artificers will remain encamped at Buena Vista. At the commencement of the disorder, I recommended our hospital being put in quarantine, and not to allow a sick person to remain any where else; also to have permanent wardmasters and orderlies, and a fatigue-party for carrying the sick and burying the dead, as we then had no carts.

"Estimate of the total strength of the garrison on the 1st of Sept. last, including the military, 12000; of these, 3000 inhabitants quitted the place shortly after the disease appeared (several of them died on board ship, having carried the contagion with them.) Since the 1st of September 5000 died, which will make the average of deaths to be more than one in two, without reckoning the number of the military that have not yet taken the disorder; these I calculate about 800. The 10th regiment have lost only two or three men of those who have been in the East Indies, and very few indeed of their recruits recover, which evidently shews that the climate has done more for them than medical aid.

"Those who have had the malignant fever of the West Indies, are also secure from this, as there are but very few, if any exceptions.

"December 15. We continue healthy, only one man confined to bed with a pectoral complaint. I had but two men admitted in the hospital with fever since the 6th, so that the men being now encamped seems to effect nothing, as as we had but one admitted four days before, and the other three days after they were ordered into quarantine.

"I am, &c.

"T. KINNING,

"Assistant Surgeon, R. Reg. Artillery."

---

*Eight Cases of the Fever which has proved so fatal at Gibraltar.*

Nov. 3, 1804, ten o'clock, A. M. Gunner David Dixey, aged about twenty-four years, dark complexion, gross habit of body, had been discharged the hospital about a fortnight, where he was eight days with sore eyes, of which he recovered, and did duty as usual; was suddenly seized this morning at Willis's camp, with chilliness, violent pains in his back and limbs, also in his left side, this last of short continuance; throat rather sore, tongue furred and whitish, head and stomach easy; had an evacuation by stool yesterday; frequent desire of making water, which was thick, high coloured, and in small quantity; pulse full and of 100 beats. Ordered an emetic of ipecac. gr. xv. antimon. tartar. gr. j. to be wrought off with two quarts of warm water.

Three o'clock, P. M. Emetic operated well, nothing particular in the matter ejected; had two copious fœtid black stools; heat rather increased, tongue the same, pains in his loins worse, stomach easy, pains in his limbs something abated, face flushed; pulse 104, rather weak. Ordered oatmeal gruel with a little wine in it.

Six o'clock. Since last report, he had one black stool, urine free, has a slight pain in his forehead, thirst, eyes rather red, tongue more furred, mouth dry; took a little tea and toast; pulse 108, moderately full. Ordered one grain of James's powder to be taken directly; and should it have no sensible effect, another in six hours.

4th. eight o'clock, A. M. The first dose of James's powder operated rather violently, both as an emetic and purgative; is now easy, but thirsty; face rather swelled; took tea and toast; is very apprehensive for his recovery; wished to drink toast and water, which I allowed him; pulse 104, moderately full, but very soft. Ordered two gills of wine to be taken in gruel or panada through the day.

Six o'clock, P. M. Took one gill of wine, also tea and toast, which he soon after vomited; head is well, his back and limbs are painful, tongue very much furred; pulse 100, rather weak; complains of chilliness, yet heat moderate.

rate. Ordered an additional gill of wine before morning; and to take a large spoonful of the following every hour: ℞. Decoct. cort. Peruv. ℥viij. tinct. ditto, ℥j. spt. æther. vit. c. ʒj. tinct. opii, gr. xl. M.

5th. eight o'clock, A. M. Slept very well; had two fœtid black stools in the night, vomited a little this morning; since then he eat some tea and toast, which as yet sit easy; some little pains in his limbs and throat; pulse 100, moderately full but feeble; half the tongue toward the tip is clear and shining. Ordered the medicines to be continued, to have four gills of wine, sago, and tapioca.

Three o'clock, P. M. Thinks himself well, is free of pain, vomited a good deal, ate about an ounce of meat, (which was given him without leave) took his medicines regularly, face rather pale, tongue not so shining, hawks some bloody slimy matter, eyes clearer; pulse 106, rather feeble; has had two gills of wine. Ordered four more, and to continue his medicine.

Seven o'clock, P. M. Vomited once, continues easy, tongue not so shining; pulse 102, rather feeble. Ordered to continue wine and medicines.

6th. eight o'clock, A. M. Passed a bad night, is delirious, one stool, vomited twice, is now rather comatose, tongue dry, is very weak, no appetite; pulse 90, very feeble. Ordered a bottle of sherry wine, and to continue his medicines, with æther. vitriol. ʒj. to ℥iv. of the bark, M.

Three o'clock, P. M. No alteration.

Seven o'clock, P. M. Vomited since about a quart of matter resembling coffee grounds; perfectly free from pain; delirious; pulse 102, very feeble. Ordered to continue his wine and medicine, and a table spoonful of water, strongly acidulated with elixir of vitriol, every half hour.

Nov. 7, eight o'clock, P. M. Slept but little, was delirious, feels quite easy, vomited black fluid very often, had hiccup for an hour nearly, took his medicines regularly, two bottles of white wine; pulse scarcely to be perceived. Ordered to continue his medicines, &c. and to apply synapisms to his feet.

Two o'clock, P. M. Is just expired; about an hour ago, he bled profusely from his mouth and nose.

By Mr. BELL, of the Garrison Staff.

Nov. 30, 1804, seven o'clock, P. M. Private Thoroughgood, of the Artificers Company, aged twenty-eight years, low stature, thin habit, dark complexion, grey eyes, usually healthy; had been employed as cook in the hospital for six six weeks; was dismissed on the 26th instant, in perfect health; was suddenly attacked to day, when at work, with an acute pain in his forehead, loins, and limbs; drank less than a pint of common wine during the morning; is restless; has thirst and nausea, eyes red, tongue clean, bowels regular, skin dry and hot; pulse 144. Ordered, pulv. ipecac. ℈j. to be taken directly, and worked off with a quart of warm water. Drink, toast and water.

Dec. 1, Morning Report. Emetic operated well; the fluid ejected very bitter and dark coloured, from the wine he had previously drank; his stomach and back are easy; his limbs were bathed in warm water, skin continues hot, head-ache and eyes the same; countenance livid and depressed; had two copious natural stools; pulse 144, softer. Ordered to take ol. Ricini ℥iij. and to apply a blistering plaster on the back of his neck. Diet, broth, gruel and tea; drink, tea and toast and water

Evening Report. The Castor oil operated seven times, is perfectly free of pain, had his limbs bathed twice, general perspiration, is inclined to sleep; pulse 130, softer. Ordered to continue drink.

Dec. 2. Had five hours sleep, six purging stools, some pain in his legs, countenance sunk, tongue shining and dry, no perspiration at present; was seized with general spasms about three o'clock this morning, and which continue to come every ten minutes; is delirious at times, and kept in bed with difficulty; pulse 100, weak and intermitting. Ordered to bathe his limbs as before, to take spt. æther. vit. c. ℨß. mist. camphor. ℥ß. every two hours; to have a clyster every four hours of extr. cinchonæ ℥iij. g. ass. fœtid. ℈j. dissolved in four ounces of beef-tea, with tinct. opii. gt. 30; to apply blistering plasters to his legs, to be removed to a fresh part every four hours, or when the skin begins to inflame.

Evening Report. The spasms have not returned since he took the first dose of his medicine; he cannot bear wine or porter; retained each injection an hour; was perfectly sensible five hours; pulse was regular during the day, and is now 120, small and feeble; difficult articulation continues since mid-day; is now delirious; difficult inspiration from an acute pain at his sternum; ate a little milk, soup and tea; passed involuntary stools. Ordered to continue æther and camphor. m. and to apply a blister plaster to his breast, and synapisms to his feet. Drink, milk and rice.

Dec. 3. Expired betwixt ten and eleven o'clock last night, in a low muttering delirium, and passing involuntary stools and urine.

The body is now completely putrid.

Nov. 5, 1804. Gunner Farmer, of the Royal Artillery, aged twenty-five years, of a dark complexion, spare habit of body, usually healthy and sober; complains of pain in his head, back and limbs, and sickness at stomach; bowels costive, eyes red and protuberant, tongue white, skin dry; says his complaint began yesterday; pulse 96, and strong. ℞. Pulv. r. ipecac. ℈j. to be taken directly, and worked off with warm water.

Evening Report. Emetic operated well; the fluid ejected of a greenish colour; head-ache and other pains continue; no stool; pulse 102, feeble. R. Ol. ricin. ℥ß. to be taken in a little peppermint water.

Nov. 6th. Morning Report. Had three evacuations, nearly natural, by stool; slept tolerably well, and perspired toward morning; head-ache and pain in his loins continue; pulse 90, and softer. To apply a blistering plaster to the nape of his neck, and to take of the pulv. Jacobi gr. ij. every two hours. Diet, gruel, broth, beef-tea, and tapioca. Drink, barley-water, toast and water, &c. in small quantities.

Evening Report. Took three doses of his medicine, which purged and vomited him; the blistering plaster was not applied, through mistake of the ward-master; perspires, though very restless; complains of coldness in his feet. Ordered to apply the blistering plaster, as directed in the morning; also pediluv. Drink, a pint of porter, a gill of wine in gruel, at stated intervals; pulse 108, soft.

Nov. 7th, Morning Report. Had a disturbed night; passed six thin natural stools; took only a wine glassful of porter and a little gruel; sickness at stomach and coldness in his feet; eyes red, tongue white, with a red seam round its edge; pulse 90, soft. Ordered opium gr. j. every two hours if the nausea continues; pediluv. Continue diet. Drink, two gills of wine before the evening.

Evening Report. Had four hours sleep; countenance depressed, as in the morning; thirsty; urine free, and deposited a cloud; took his wine; skin moist; pulse 102, soft; had occasion to take only one pill; no stool since morning. Drink, a pint of porter to-night.

Nov. 8th, Morning Report. Had a good night; countenance more lively; perspires; complains of a heaviness at at stomach; perfectly free of pain; had a free natural stool this morning; tongue white; pulse 72, soft. Continue diet. Drink, two gills of wine.

Evening Report. Was restless to-day; had no sleep; urine high coloured; took half his wine; no appetite; heaviness at stomach continues, but no reaching; pulse 84, soft. Ordered, the remainder of his wine; also a common clyster.

Nov. 9th, Morning Report. Slept tolerably well; is free of pain; stomach easy; had one evacuation after the injection; pulse 84, undulating; skin moist; tongue white; no appetite. Ordered, diet, a high-seasoned beef-steak. Drink, two gills of wine.

Evening Report. Had a good day; sat up in bed while he took a little of his diet; stomach easy; pulse 96, soft; no stool; tongue nearly natural. Ordered the remainder of his wine; also a common injection.

Nov. 10th, Morning Report. Had a good night; three evacuations after the injection; pulse 84, soft; tongue clean. Diet, seasoned solid food. Drink, two gills of wine.

Evening Report. Is convalescent.

11th Report. Continues free of fever.

13th Report. Strength returning rapidly.

15th Report. Discharged cured.

November 29, 1804. Gunner Thomas Kinland, of the Royal Artillery, aged thirty years, usually sober, of middle size, brown complexion and light eyes, lately discharged from the hospital, recovered from an abscess, for which he had taken a good deal of bark; complains of nausea, pain in the head, back and limbs, debility, restlessness; bowels regular, skin hot, tongue white, pulse 90, hard. Ordered, pulv. rad. ipecac. ℈i. to be taken directly. Diet, tea, gruel, sago, broth, &c. Drink, toast and barley water.

30th. Emetic operated well, one stool; a blistering plaster on the back of his neck has relieved his head-ache, also his pains in general are abated; pulse and skin as yesterday. Ordered, of ol. Ricini ℥ß. to be taken immediately. Diet to be continued with a small quantity of wine in his gruel; continue drink.

December 1. Seems considerably better, pulse and skin natural; perspired in the night; tongue cleaner; feels a return of appetite; had three copious good evacuations. Ordered, diet to be continued; drink, a gill of wine.

December 2. Passed a good day and night; ate moderately; he got up this morning being tired of bed, and in fifteen minutes after vomited a pint of brown fluid: his face is now pale; feels great debility; pulse 76, soft; bowels regular, appetite gone, tongue clean and moist, perfectly free of pain; a nausea continues. Ordered, spt. æther. vit. ʒß. and afterwards a pill of Cayenne pepper of gr. 4, every two hours; to have a clyster of extr. cinchonæ ʒiij. tinct. opii ℈i. in a little beef tea every three hours. Should not he find relief in six hours, to apply a blistering plaster to his stomach, to discontinue the Cayenne pills, and to take æther and opium, gr. j.

Evening Report. He retained each of the clysters nearly two hours. The Cayenne pills induced more vomiting of a darker cast; took two opium pills at an interval of two hours, and applied a blistering plaster, which seem to have checked the vomiting; pulse 104, feeble; countenance more lively; extremities rather cold; had hiccup the greater part of the day, which has also abated since he took the opium. Ordered to continue the opium and æther if more vomiting ensues, if he can bear light nutriment with it. To take an ounce of water strongly acidulated with elix. of vitriol every hour; to have three injections of the extr. cinchon. as before; to apply frictions with a warm flannel to his hands and feet, and warm bricks to the latter. Diet, milk and rice; drink, mulled wine, a spoonful very often, (every ten minutes.)

December 3, Morning Report. He had four hours sleep in the night; hiccup and vomiting are just returned; great debility, extremities moderately warm; one fœtid stool; thirst; he cannot bear wine, porter, or spirits in any way; pulse 96, soft; skin and tongue natural; in countenance depressed; perfectly collected. Ordered, a clyster every two hours of rich beef tea ℥iv.; continue the elix. vitr. every half hour; op. gr. jß. if the symptoms do not abate in two hours; continue diet; drink only to wet his mouth.

Evening Report. Had three doses of opium since; took the elix.; the hiccup, &c. returned at intervals; he could retain the clysters but a very short time; skin cool, pulse 84, stronger; countenance depressed; thirst, extremities cold and livid. Ordered, to apply warm bricks to his feet; to take opium, a grain every two hours, and spt. æther. vitr. (a tea spoonful in a little camphor mixt. every hour.) Diet as before; drink, elix. vitr. diluted.

December 4, Morning Report. Vomited frequently; continues hiccup when awake; slept at intervals; directions respecting respecting medicine were strictly attended to; tongue, skin, and belly regular; countenance the same; boiled milk sits easier than any other kind of food; drank nearly two gills of wine. Ordered, æther every half hour; continue opium; clysters every three hours of extr. cinchona ℥ij. tinct. opii gr. x. in beef tea.

Evening Report. No vomiting since ten o'clock this morning; he would not take any pills; had his æther and drink, the former every hour since two o'clock; had four injections, the two last remained with him; since four o'clock got much worse, a low muttering delirium generally; at whiles laughs, and at other times calls out in the most excruciating agony; tongue clean; breath exceedingly offensive; body naturally warm; extremities cold; articulation distinct; hiccup almost incessant till within the last hour; blisters were applied to his legs, and cataplasms to his feet; countenance sunk; pulse imperceptible. Ordered, to continue æther with the addition of spt. lavender c. and spt. ammmon c. mulled wine and the diluted elix. alternately for drink; clysters of milk.

December 5, Morning Report, six o'clock. He expired at ten o'clock last night; hiccup and vomiting did not return. The body is now quite putrid.

November 13. Gunner William Doyle of the Royal Artillery, fair complexion, low stature, aged about 28 years, employed as an officer's servant, had been lately in the hospital with ophthalmia, of which he was cured; his character a good sober man; he did not take the prevailing malignant fever while in the hospital, as those with ophthalmia were separated to a different part of the house, to prevent communication as far as possible; complained of head-ache, pain in his back and limbs, nausea; pulse 120, quick and firm, skin hot, belly costive. Ipecac. ℈j. to be taken directly.

14th. The emetic operated well; feels relieved, excepting the head-ache; skin moist, tongue white, pulse 80, soft; one small evacuation by stool. Ordered, a blistering plaster to the back of his neck, and a purging clyster; to drink toast and water, porter and water for common drink in small quantities; diet, panada, gruel, broth and tea.

15th. Passed the day tolerably easy, blister rose well; had a restless night; took two gills of porter, and a little of the diet prescribed; is now uneasy at the stomach, loaths his food and drink; had two evacuations by the injection; jection; pulse 84, soft, skin nearly natural. Ordered, a starch clyster with tinct. opii ʒi. also æther. vitr. c. ʒß. spt. lavend. c. ℈j. to be taken in a little water, when uneasy at his stomach; to bathe his feet in warm water. Diet, beef tea, tapioca; drink, thin mucilage of g. arabic, and toast and water in small quantity

16th. Passed the day tolerably easy; found relief from two doses of his medicine, taken at an interval of two hours: toward evening the nausea returned; in the night vomited twice, matter like coffee grounds; he took no wine or porter; had his medicine three times during the night; pulse 96, and soft; had two fœtid evacuations by stool; complains of great debility; disturbed sleep. Ordered, ol. Ricini ʒiij. afterwards the æther and spt. lavend. c. every two hours; diet, panada, rice, gruel and beef tea; drink, rice water and four gills of wine, a small quantity as above.

17th. Since last report he vomited four times fluid of a darker colour; had a free stool and more natural; inclined to sleep; countenance depressed, pulse 80, soft. He took but a small quantity of wine, and that mulled; urine high coloured, without sediment, and in small quantities; skin natural; free of pain. Ordered, opium, gr. j. every four hours until relieved of the nausea; to discontinue the æther mixture. Diet, bread and milk boiled; drink, four gills of wine, rum, or brandy with water.

18th. He took but three pills; after the first he vomited but once; feels greatly relieved; debility continues; had several hours composed sleep; he could not bear spirits in any way; took a little wine, also a little bread and milk; loathes any other food when offered him; pulse firmer, countenance more lively, bowels more natural, skin cool, tongue clean. Ordered, æther ʒß. in a little drink; diet, milk; drink, wine two gills.

19th. Continues easy; took two doses of æther, also a little of his diet, but cannot relish wine or porter. Pulse, tongue, skin, and bowels natural; continue milk diet, also ordered a beef steak well seasoned; drink toast and rice water.

20th. Continues better but weak; ate a little of the steak; took milk morning and evening.

21st. Continues better; sat up in his bed at his meals; still loathes wine, &c. To continue his diet, also ordered a bit of fowl.

22d. Is now able to sit up longer in bed; continue diet.

23d. Has

23d. Has now gained some appetite, also strength; to continue his diet; drink, porter a bottle.

24th. Yesterday ate hearty of fowl, and drank his porter in the course of the day; is now convalescent; continue diet, &c.

25th. Continues better; is removed to a healthy ward; continue diet and porter.

26th. Is gaining strength; diet, hashed meat; drink, porter.

27th. Feels strong, and wishes to be discharged; diet and drink whatever he wishes.

29th. Discharged cured.

Nov. 5, 1804, six o'clock, P. M. Bomb. Morton, of the Royal Artillery, aged 28 years, fair complexion, full habit, and tall, of good character; had an attack of fever in September last, of which he soon recovered, and did duty for a month; complains of sickness at his stomach, headache, pains in his limbs, thirst; tongue white, bowels costive. He says his illness began last night; pulse 104, firm; ℞ Pulv. rad. ipecac. ℈i. to be taken directly, and after the operation to bathe his feet in warm water.

6th. Morning Report. Emetic operated well, and also purged him nine times; had a bad night, his head giddy, free of pain; urine clear and in proper quantity; breathing easy; skin dry; pulse 104, soft. Ordered, a blistering plaster to the back of his neck; diet, gruel, beef tea, rice water; drink, one gill of wine, toast and water.

Evening Report. Was restless, though free of pain; skin hot and dry; face flushed; eyes continue red and very protuberant; passed one stool streaked with blood; his articulation difficult; memory impaired; pulse 96, soft. Ordered, a pint of porter to-night; pediluv. and a small clyster of broth.

7th. Morning Report. Had a good night; one natural stool; took a little of his porter; eyes and articulation the same; urine turbid; countenance darker; skin cooler, but without perspiration; pulse 96, soft. Ordered, mulled wine two gills, a little of it to be given frequently; to apply frictions with warm flannels to his extremities; diet, tapioca, gruel, and gravy soup.

Evening Report. Countenance depressed and livid; sleeps almost constantly; breathing easy; passed no stool or urine since morning; took nearly a gill of wine and some of his diet; extremities cold; free of pain; pulse 108, small and soft. Ordered, a clyster of broth every two hours; blisters ters to his legs and mustard poultices to his feet; ℞. Spt. æther. vitr. c. gr. 30, M. camphor ℥ß. to be taken every hour; drink porter

8th. Morning Report. Slept well in the night; countenance depressed; passed one involuntary stool; articulation and swallowing more difficult; the blisters did not rise; pulse 106, soft. Ordered, wine and porter as much as possible; to apply warm bricks to his feet; continue æther and camph. mixt.; to have a clyster every two hours of ext. cort. peruv. ʒiij. dissolved in a gill of beef tea.

Evening Report. Countenance perfectly livid; extremities cold; passed two involuntary stools, one previous to his injections; took his medicine, but scarcely any thing else; had four clysters administered; lays totally insensible; pulse imperceptible. Ordered, to continue his medicine, and any nutriment he can swallow.

9th. Morning Report, seven o'clock. He expired at eleven o'clock last night. The body is so putrid and offensive, it is not considered advisable to examine any internal part.

Nov. 15, 1804. Gunner Michael Boyle, of the Royal Artillery, a married man, usually sober, and of good character, of a weak habit of body, fair complexion, light blue eyes, low stature, generally enjoying good health, had been lately in the hospital for ophthalmia, afterwards for a slight fever; pulse 112, firm; skin dry. Complains of head-ache, nausea, pains all over the body and limbs, restlessness, eyes red and protuberant, thirst, white tongue, bowels costive. Ordered, ipecac. ℈j. to be worked off with warm water; afterwards to apply a blistering plaster to the nape of the neck. Diet, panada, tapioca, sago, broth, tea, &c.

16th. Emetic operated well, the blister rose well; had an injection in the evening without salt, had six stools in the night, at intervals slept easy; skin rather moist, head-ache rather abated, tongue furred, surrounded with a red seam, bowels uneasy, countenance lively. Ordered, a starch clyster. Diet, as before; drink, two gills of wine, toast and rice water; pulse 100, softer.

17th. Rested ill in the night; frequent watery stools; stomach easy, but no relish for food; pulse 96, weaker; skin, a sensation of heat remained after the touch; no perspiration, pains gone, urine free, no sediment. Ordered, tinct. opii & spt. vit. c. gr. x. after every stool. Continue diet and drink.

18th. Was

18th. Was restless; passed seven watery stools; countenance depressed, and of a livid appearance; eat little; took his wine; pulse 90, soft; complains of great debility; disturbed sleep; skin cool, tongue white, rather shining. Ordered a starch clyster with tinct. opii ʒj. Diet as before; drink, half a bottle of porter and two gills of wine.

19th. Feels relieved; had only one evacuation by stool, nearly natural; slept soundly in the night; perspired freely; urine free, and no sediment; pulse 80, soft; countenance depressed. Continue diet and drink, and brandy and water.

20th. Rested ill in the night; had one natural stool; countenance continues livid, eyes red, tongue clean, extremities cold; could not bear spirits in any way; took a little of his diet and drink; pulse 86, feeble. Ordered the camphor mixture, two spoons full every two hours. Diet as before; also a beef steak well seasoned. Drink one bottle of porter and two gills of wine.

21st. He took his medicine four times, feels confidence and is better, no stool, stomach, &c. easy; pulse 80, soft; eat a little, took his drink, but cannot relish more porter; extremities warm; had some hours comfortable sleep. Continue M. camph. Diet as before and potatoes. Drink, six gills of wine.

22d. Is considerably better, countenance lively, pulse natural; had one stool; skin cool; took a bit of steak; had his medicine three times, took his drink; discontinue medicines. Continue diet and drink.

23d. Is better, rested well; one stool; pulse and skin natural. Ordered diet, fowl and gravy soup. Drink, six gills of wine.

24th. Took his diet and drink with appetite; is convalescent. Ordered diet, fowl and gravy soup. Drink, half a bottle of porter and six gills of wine; is ordered to a healthy ward.

25th. As at last Report. Continue diet and drink.

26th. Continues better, and has gained strength. Continue diet. Drink, half a bottle of porter and four gills of wine.

27th. Continues gaining strength. Diet, hashed meat. Drink, one bottle of porter.

30th. Convalescent. Discharged cured.

Nov. 9, 1804. Morning Report. Private Bloomfield of the R. M. Artificers, aged twenty years, of a fair complexion and low stature, usually healthy, had been in hospital about a month since with a slight fever; complains of of pain in his head, back, and limbs; prostration of strength; sickness of stomach; pulse 78, firm; tongue white, skin dry; felt his illness come on yesterday; had a purging stool this morning. ℞. Pulv. rad. ipecac. ℈j.

Evening Report. Emetic operated well; pains continue; no stool; skin moist; pulse 80, softer; tongue white, with a red seam round its edge; slept three hours. Ordered, a common clyster, also a blistering plaster to the back of his neck.

10th. Morning Report. Had seven stools; restless; nausea; hæmorrhage from his nose; no appetite; head not so painful; countenance lively; tongue as yesterday; pulse 84, soft. Diet prescribed, sago, gravy soup; to drink a gill of wine in his food. Drink rice water, and toast and water.

Evening Report. Uneasy at stomach, particularly after taking food; five stools; frequent retching, but without rejecting any thing from his stomach. Ordered, to drink camomile water to produce vomiting; afterwards, ℞. Tinct. opii & spt. æther. vit. c. ā gt. x. in peppermint water.

11th. Morning Report. Took five doses of his medicine, which did not induce sleep or relieve the retching; had two stools; when he attempts to turn on his right side it induces full vomiting; continues to discharge blood from his nose and mouth; heaviness and also pain at stomach; pulse 70, soft. Ordered, a blistering plaster to his stomach. Diet as before and tapioca. Drink mucilage of g. arab.

Evening Report. Slept some; blister rises well; more easy at stomach; one stool; pulse 72, soft; skin cool. Ordered, a gill of wine. Continue mucilage.

12th. Morning Report. Is easier; slept a little in the night; had two stools; thirst; perspired a little; pulse 75, soft. Ordered, drink and diet as before.

Evening Report. Restless; no sickness at stomach; two stools; pulse 72, soft. Ordered, to continue mucilage and wine.

13th. Morning Report. Passed a bad night; no stool; his nose bleeds a little; pulse 70, feeble. ℞. Mixt. camphor. ℥ß. every two hours. Diet as before. Drink, a bottle of porter, and two gills of wine in his food.

14th. Slept tolerably; could not relish his porter; one stool; gums bleed; eyes of a yellow tinge; the blistered part on his neck fœtid; pulse 78, soft; countenance depressed. Ordered, diet a well-seasoned beef steak and potatoes. Drink, four gills of wine, as he can bear it; also the decoct. cinch. with the acid. vit. to gargle his mouth.

16th. Continued his plan of cure; is considerably better but very weak; hæmorrhage from the gums entirely stopped; stopped; feels a return of appetite. Ordered, diet solid animal food. Drink, six gills of wine.

18th. Continues better; bowels regular; tongue clean; eyes losing the yellow tinge. Ordered, to continue his diet and wine, ad libit. also to be removed to a healthy ward.

25th. Continues convalescent; had followed his plan of diet and wine, only diminishing the quantity of the latter as he gained strength. Discharged cured.

